# The ASRG database: identification and survey of *Arabidopsis thaliana *genes involved in pre-mRNA splicing

**DOI:** 10.1186/gb-2004-5-12-r102

**Published:** 2004-11-29

**Authors:** Bing-Bing Wang, Volker Brendel

**Affiliations:** 1 Department of Genetics, Development and Cell Biology Iowa State University, Ames, IA 50011-3260, USA; 2 Department of Statistics, Iowa State University, Ames, IA 50011-3260, USA

## Abstract

A total of 74 small nuclear RNA (snRNA) genes and 395 genes encoding splicing-related proteins were identified in the *Arabidopsis *genome by sequence comparison and motif searches, including the previously elusive *U4atac *snRNA gene. Most of the genes have not been studied experimentally. Classification of these genes and detailed information on gene structure, alternative splicing, gene duplications and phylogenetic relationships are made accessible as a comprehensive database of *Arabidopsis *Splicing Related Genes (ASRG) on our website.

## Abstract 2

The database of *Arabidopsis *splicing related genes includes classification of genes encoding snRNAs and other splicing related proteins, together with information on gene structure, alternative splicing, gene duplications and phylogenetic relationships.

## Rationale

Most eukaryotic genes contain introns that are spliced from the precursor mRNA (pre-mRNA). The correct interpretation of splicing signals is essential to generate authentic mature mRNAs that yield correct translation products. As an important post-transcriptional mechanism, gene function can be controlled at the level of splicing through the production of different mRNAs from a single pre-mRNA (reviewed in [1]). The general mechanism of splicing has been well studied in human and yeast systems and is largely conserved between these organisms. Plant RNA splicing mechanisms remain comparatively poorly understood, due in part to the lack of an *in vitro *plant splicing system. Although the splicing mechanisms in plants and animals appear to be similar overall, incorrect splicing of plant pre-mRNAs in mammalian systems (and vice versa) suggests that there are plant-specific characteristics, resulting from coevolution of splicing factors with the signals they recognize or from the requirement for additional splicing factors (reviewed in [2,3]).

Genome projects are accelerating research on splicing. For example, with the majority of splicing-related genes already known in human and budding yeast, these gene sequences were used to query the *Drosophila *and fission yeast genomes in an effort to identify potential homologs [4,5]. Most of the known genes were found to have homologs in both *Drosophila *and fission yeast. The availability of the near-complete genome of *Arabidopsis thaliana *[6] provides the foundation for the simultaneous study of all the genes involved in particular plant structures or physiological processes. For example, Barakat *et al. *[7] identified and mapped 249 genes encoding ribosomal proteins and analyzed gene number, chromosomal location, evolutionary history (including large-scale chromosomal duplications) and expression of those genes. Beisson *et al. *[8] catalogued all genes involved in acyl lipid metabolism. Wang *et al. *[9] surveyed more than 1,000 *Arabidopsis *protein kinases and computationally compared derived protein clusters with established gene families in budding yeast. Previous surveys of *Arabidopsis *gene families that contain some splicing-related genes include the DEAD box RNA helicase family [10] and RNA-recognition motif (RRM)-containing proteins [11]. At present, the *Arabidopsis *Information Resource (TAIR) links to more than 850 such expert-maintained collections of gene families [12].

Here we present the results of computational identification of potentially all or nearly all *Arabidopsis *genes involved in pre-mRNA splicing. Recent mass spectrometry analyses revealed more than 200 proteins associated with human spliceosomes ([13-17], reviewed in [18]). By extensive sequence comparisons using known plant and animal splicing-related proteins as queries, we have identified 74 small nuclear RNA (snRNA) genes and 395 protein-coding genes in the *Arabidopsis *genome that are likely to be homologs of animal splicing-related genes. About half of the genes occur in multiple copies in the genome and appear to have been derived both from chromosomal duplication events and from duplication of individual genes. All genes were classified into gene families, named and annotated with respect to their inferred gene structure, predicted protein domain structure and presumed function. The classification and analysis results are available as an integrated web resource, the database of *Arabidopsis *Splicing Related Genes (ASRG), which should facilitate genome-wide studies of pre-mRNA splicing in plants.

## ASRG: a database of *Arabidopsis *splicing-related genes

Our up-to-date web-accessible database comprising the *Arabidopsis *splicing-related genes and associated information is available at [19]. The web pages display gene structure, alternative splicing patterns, protein domain structure and potential gene duplication origins in tabular format. Chromosomal locations and spliced alignment of cognate cDNAs and expressed sequence tags (ESTs) are viewable via links to the *Arabidopsis *genome database AtGDB [20], which also provides other associated information for these genes and links to other databases. Text-search functions are accessible from all the web pages. Sequence-analysis tools including BLAST [21] and CLUSTAL W [22] are integrated and facilitate comparison of splicing-related genes and proteins across various species.

## *Arabidopsis *snRNA genes

A total of 15 major snRNA and two minor snRNA genes were previously identified experimentally in *Arabidopsis *[23-28]. These genes were used as queries to search the *Arabidopsis *genome for other snRNA genes. A total of 70 major snRNAs and three minor snRNAs were identified by this method. In addition, a single *U4atac *snRNA gene was identified by sequence motif search. We assigned tentative gene names and gene models as shown in Table 1, together with chromosome locations and similarity scores relative to a representative query sequence. The original names for known snRNAs were preserved, following the convention atUx.y, where x indicates the U snRNA type and y the gene number. Computationally identified snRNAs were named similarly, but with a hyphen instead of a period separating type from gene number (atUx-y). Putative pseudogenes were indicated with a 'p' following the gene name. Pseudogene status was assigned to gene models for which sequence similarity to known genes was low, otherwise conserved transcription signals are missing and the gene cannot fold into typical secondary structure. A recent experimental study of small non-messenger RNAs identified 14 tentative snRNAs in *Arabidopsis *by cDNA cloning ([29], GenBank accessions 22293580 to 22293592 and 22293600, Table 1). All these newly identified snRNAs were found in the set of our computationally predicted genes.

### Conservation of major snRNA genes

As shown in Table 1, each of five major snRNA genes (U1, U2, U4, U5 and U6) exists in more than 10 copies in the *Arabidopsis *genome. U2 snRNA has the largest copy number, with a total of 18 putative homologs identified. Both U1 and U5 snRNAs have 14 copies, U6 snRNA has 13 copies, and U4 snRNA has only 11 copies. Sequence comparisons within *Arabidopsis *snRNA gene families showed that the U6 snRNA genes are the most similar, and the U1 snRNA genes are the most divergent. Eight active U6 snRNA copies are more than 93% identical to each other in the genic region, whereas active U1 snRNAs are on average only 87% identical. The U2 and U4 snRNAs are also highly conserved within each type, with more than 92% identity among the active genes. Details about the individual snRNAs and the respective sequence alignments are displayed at [30].

Previous studies identified two conserved transcription signals in most major snRNA gene promoters: USE (upstream sequence element, RTCCACATCG (where R is either A or G) and TATA box [24-27]. All 14 U5 snRNAs have the USE and TATA box. Furthermore, their predicted secondary structures are similar to the known structure of their counterparts in human, indicating that all these genes are active and functional (structure data not shown; for a review of the structures of human snRNAs, see [31]). Similarly, we identified 17 U2, 10 U1, nine U4, and nine U6 snRNA genes as likely active genes, with a few additional genes more likely to be pseudogenes because of various deletions. U4-10 and U6-7 do not have the conserved USE in the promoter region, but their U4-U6 interaction regions (stem I and stem II) are fairly well conserved. U2-16 is also missing the USE but has a secondary structure similar to other U2 snRNAs. These genes may be active, but differences in promoter motifs suggest that their expression may be under different control compared with other snRNAs homologs. The U2-17 snRNA has all conserved transcription signals, but 20 nucleotides are missing from its 3' end. The predicted secondary structure of U2-17 is similar to that of other U2 snRNAs, with a significantly shorter stem-loop in the 3' end as a result of the deletion. We are not sure if the U2-17 snRNA is functional, but the conserved transcription signals imply that it may be active.

Other conserved transcription signals were also identified in most active snRNAs, including the sequence element CAANTC (where N is either A, C, G or T) in U2 snRNAs (located at -6 to -1) [23], and the termination signal CAN_3-10_AGTNNAA in U snRNAs (U1, U2, U4 and U5) transcribed by RNA polymerase II (Pol II) [23,24,32]. The previously identified monocot-specific promoter element (MSP, RGCCCR, located upstream of USE) in U6.1 and U6.26 [33] is also found in five other U6 snRNA genes (U6.29, U6-2, U6-3, U6-4, U6-5). In all seven U6 snRNAs the consensus MSP sequence extends by two thymine nucleotides to RGCCCRTT. Although the MSP does not contribute significantly to U6 snRNA transcription initiation in *Nicotiana plumbaginifolia *protoplasts [33], the extended consensus may imply a role in gene expression regulation in *Arabidopsis*.

### Low copy number of minor snRNA genes

The minor snRNAs are functional in the splicing of U12-type (AT-AC) introns. Four types of minor snRNAs, which correspond to four types of major snRNAs, exist in mammals. U11 is the analog of U1, U12 is the analog of U2, U4atac is the analog of U4, and U6atac is the analog of U6. The U5 snRNA seems to function in both the major and minor spliceosome [34]. Two minor snRNAs (atU12 and atU6atac) were experimentally identified in *Arabidopsis *[28]. Both have the conserved USE and TATA box in the promoter region. We identified another *U6atac *gene (*atU6atac-2*) by sequence mapping. This gene has a USE and a TATA box in the promoter region. The *atU6atac-2 *gene is more than 90% similar to *atU6atac *in both its 5' and 3' ends, with a 10-nucletotide deletion in the central region. The putative U4atac-U6atac interaction region in atU6atac-2 is 100% conserved with the interaction region previously identified in atU6atac [28,35].

U11 and U4atac have not been experimentally identified in *Arabidopsis*. BLAST searches using the human U11 and U4atac homologs as queries against the *Arabidopsis *genome failed to find any significant hits, indicating divergence of the minor snRNAs in plants and mammals. Using the strategy described below, we successfully identified a putative *Arabidopsis U4atac *gene. It is a single-copy gene containing all conserved functional domains. We also found a single candidate U11 snRNA gene (chromosome 5, from 17,492,101 to 17,492,600) that has the USE and TATA box in the promoter region. This gene also contains a putative binding site fr Sm protein and a region that could pair with the 5' splice site of the U12-type intron.

### Identification of an *Arabidopsis U4atac *snRNA gene

**Figure 1:**
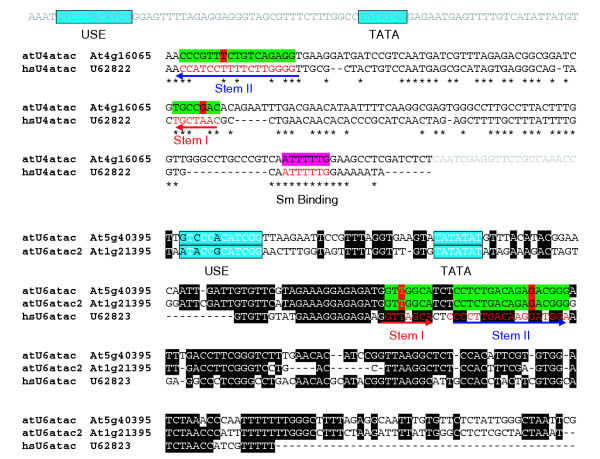
Sequence alignments of U4atac and U6atac snRNAs. The tentative *Arabidopsis *U4atac snRNA was aligned against the human U4atac snRNA (U62822) using CLUSTAL W [22]. Possible sequence domains are indicated by different background colors, with cyan indicating transcription signals (USE, upstream sequence element; TATA, TATA box), green indicating the region involved in the stem-loop-stem structure, and pink indicating the domain that binds Sm proteins. The corresponding interaction region in U6atac snRNA is also marked in green. Red background indicates G-T base-pairs in the stem-loop structure. Grey letters indicate the genome sequence upstream and downstream of the putative U4atac gene. Asterisks (upper panel) and black shading (lower panel) show conserved positions in the alignment.

Like U4 snRNA and U6 snRNA, human U4atac and U6atac snRNAs interact with each other through base pairing [36]. The same interaction is expected to exist between the *Arabidopsis *homologs. Therefore, we deduced the tentative AtU4atac stem II sequence (CCCGTCTCTGTCAGAGGAG) from AtU6atac snRNA and searched for matching sequences in the *Arabidopsis *genome. Hit regions together with flanking regions 500 base-pairs (bp) upstream and 500 bp downstream were retrieved and screened for transcription signals (USE and TATA box). One sequence was identified that contains both the USE and TATA box in appropriate positions, as shown in Figure 1.

The tentative *U4atac *snRNA gene contains not only the stem II sequence, but also the stem I sequence that presumably base-pairs with U6atac snRNA stem I. Furthermore, a highly conserved Sm-protein-binding region exists at the 3' end. The predicted secondary structure is nearly identical to hsU4atac, with a relative longer single-stranded region (data not shown). With the highly conserved transcriptional signals, functional domains and secondary structure, this candidate gene is likely to be a real U4atac snRNA homolog. We named it AtU4atac and assigned At4g16065 as its tentative gene model because it is located between gene models At4g16060 and At4g16070 on chromosome 4.

### Tandem arrays of snRNAs genes

**Figure 2:**
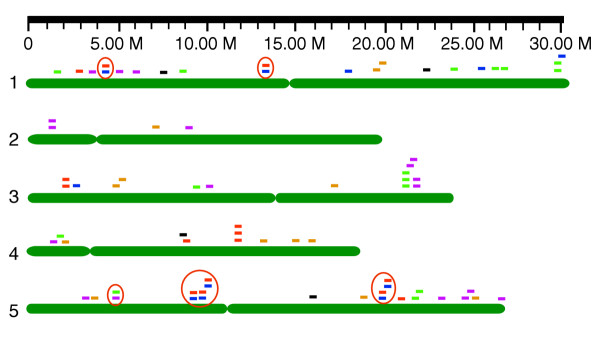
Chromosomal locations of *Arabidopsis *snRNAs. Chromosomes 1 to 5 are represented to scale by the long thick lines in dark green. The small bars above the chromosomes indicate the presence of an snRNA gene in that region. Different colors represent different snRNA types: red, U1 snRNA; magenta, U2 snRNA; blue, U4 snRNA; green, U5 snRNA; yellow, U6 snRNA; black, minor snRNA. The seven U1-U4 snRNA gene clusters (red-blue) and the single U2-U5 snRNA gene cluster (magenta-green) are indicated by red circles.

Some snRNAs genes exist as small groups on the *Arabidopsis *chromosomes [6]. We identified 10 snRNA gene clusters: seven U1-U4 snRNA clusters, one U2-U5 snRNA cluster, and a tandem duplication for both U2 snRNA (U2-10) and U5 snRNA (U5.1) (Figure 2). All seven *Arabidopsis *U1-U4 clusters have the U1 snRNA gene located upstream of the U4 snRNA gene, with a 180-300-nucleotide intergenic region. Five of the U1-U4 arrays are located on chromosome 5 (U1a/U4.1, U1-4/U4-5, U1-8/U4-7, U1-9/U4-8, and U1-13p/U4.3p), and the remaining two on chromosome 1 (U1-10/U4-6 and U1-14p/U4-10). The U2-17 and U5-10 occur in tandem array on chromosome 5, separated by fewer than 200 nucleotides.

## *Arabidopsis *splicing-related protein-coding genes

Most of the proteins involved in splicing in mammals and *Drosophila *are known [4,37,38]. In addition, recent proteomics studies revealed many novel proteins associated with human spliceosomes (reviewed in [18]). Using all these animal proteins as query sequences, we identified a total of 395 tentative homologs in *Arabidopsis*. Sequence-similarity scores and comparison of gene structure and protein domain structure were used to assign the genes to families. Each gene was assigned a tentative name based on the name of its respective animal homolog. Different homologs within a gene family were labeled by adding an Arabic number (1, 2, and so on) to the name. Close family members with similar gene structure were indicated by adding -a, -b, and -c to the name. The 395 genes were classified into five different categories according to the presumed function of their products. Ninety-one encode small nuclear ribonucleoprotein particle (snRNP) proteins, 109 encode splicing factors, and 60 encode potential splicing regulators. Details of EST evidence, alternative splicing patterns, duplication sources and domain structure of these genes are listed in Table 2. We also identified 84 *Arabidopsis *proteins corresponding to 54 human spliceosome-associated proteins. The remaining 51 genes encode proteins with domains or sequences similar to known splicing factors, but without enough similarity to allow unambiguous classification. These two categories are not discussed in detail here, but information about these genes is available at our ASRG site [39].

### The majority of snRNP proteins are conserved in *Arabidopsis*

There are five snRNPs (U1, U2, U4, U5 and U6) involved in the formation of the major spliceosome, corresponding to five snRNAs. Five snRNPs (U1 snRNP, U2 snRNP, U5 snRNP, U4/U6 snRNP and U4.U6/U5 tri-snRNP) have been isolated experimentally in yeast or human [40-45]. Each snRNP contains the snRNA, a group of core proteins, and some snRNP-specific proteins. Most of these proteins are conserved in *Arabidopsis*. All U snRNPs except U6 snRNP contain seven common core proteins bound to snRNAs. These core proteins all have an Sm domain and have been called Sm proteins. The U6 snRNP contains seven LSM proteins ('like Sm' proteins). Another LSM protein (LSM1) is not involved in binding snRNA (reviewed in [46]).

As shown in Table 2, all Sm and LSM proteins have homologs in *Arabidopsis*, and eight of them are duplicated. It is likely that these genes existed as single copies in the ancestor of animals and plants, but duplicated within the plant lineage. Only one of the 24 genes (*LSM5*, At5g48870) has been characterized experimentally in *Arabidopsis*. The *LSM5 *gene was cloned from a mutant supersensitive to ABA (abscisic acid) and drought (*SAD1 *[47]). *LSM5 *is expressed at low levels in all tissues and its transcription is not altered by drought stress [47]. cDNA and EST evidence exist for all other core protein genes, indicating that all 24 genes are active.

There are 63 *Arabidopsis *proteins corresponding to the 35 snRNP-specific proteins used as queries in our genome mapping. Very few of them have been characterized experimentally, including U1-70K, U1A and a tandem duplication pair of SAP130 [48-50]. *U1-70K *was reported as a single-copy essential gene. Expression of *U1-70K *antisense transcript under the *APETALA3 *promoter suppressed the development of sepals and petals [51]. We identified an additional homolog of *U1-70K *(At2g43370) and named it *U1-70K2*. The U1-70K2 proteins showed 48% similarity to the U1-70K protein according to Blast2 results. Both genes retain the sixth intron in some transcripts, a situation which would produce truncated proteins [48]. Interestingly, we found that five of the 10 *Arabidopsis *U1 snRNP proteins, including the U1-70K-coding genes, may undergo alternative splicing.

Several genes in U2, U5, U4/U6 and U4.U6/U5 snRNPs, but none in U1 snRNP, occur in more than three copies in the *Arabidopsis *genome. The atSAP114 family has five members, including two that occur in tandem (*atSAP114-1a *and *atSAP114-1b*). Three members have EST/cDNA evidence (Table 2). Interestingly, the predicted atSAP114p (At4g15580) protein contains a RNase H domain at the amino-terminal end, and thus *atSAP114p *shares similarity to At5g06805, a gene annotated as encoding a non-LTR retroelement reverse transcriptase-like protein. It is likely that the *atSAP114p *gene is a pseudogene that originated by retroelement insertion. There are three copies of the gene for the tri-snRNP 65 kilodalton (kDa) subunit, which are clustered on chromosome 4. Both the U4/U6 90 kDa protein and the U4/U6 15.5 kDa protein also have three gene copies, and the 116 kDa and 200 kDa subunits in U5 snRNP have four copies apiece.

The yeast U1 snRNP contains several specific proteins that are not present in mammalian U1 snRNPs [52]. As in mammals, *Arabidopsis *also lacks homologs of Prp42, a component of U1 snRNP in yeast [53]. However, *Arabidopsis *has two copies of the gene for Prp39, which are similar to Prp42. Furthermore, *atPrp39a *can produce a shorter protein isoform with a novel amino-terminal sequence by exon skipping. It is possible that the duplicates and alternative isoforms of plant U1 snRNP proteins are functional homologs of the yeast-specific proteins.

Several proteins specific to the minor spliceosome are also conserved in *Arabidopsis*. The human 18S U11/U12 snRNP contains several proteins found in U2 snRNP as well as seven novel proteins [14]. Four of the seven U11/U12-specific proteins (U11/U12-35K, 25K, 65K and 31K) are conserved in *Arabidopsis*, while the remaining three (59K, 48K and 20K) have no clear homologs. Interestingly, all four *Arabidopsis *genes are single copy in the genome, and three of them are apparently alternatively spliced (Table 2).

### Splicing factors are slightly different in *Arabidopsis *than in other organisms

We divided the splicing factors into eight subgroups according to recent human spliceosome studies [13,14,16,18]: splice-site selection proteins; SR proteins; 17S U2 associated proteins; 35S U5 associated proteins; proteins specific to the BΔU1 complex; exon junction complex (EJC) proteins; second-step splicing factors and other known splicing factors. We focused our analysis on the first two subgroups because their functions in splicing are well established. A total of 109 proteins in *Arabidopsis *were identified, corresponding to 67 human queries from all eight subgroups. Most of the proteins are conserved among eukaryotes, but some human proteins have no obvious homologs in the *Arabidopsis *genome, and some novel splicing factors appear to exist in *Arabidopsis*. About 43% of genes encoding splicing factors are duplicated in the genome, whereas some proteins, such as SF1/BBP (branchpoint-binding protein, which facilitates U2 snRNP binding in fission yeast [54]) and cap-binding proteins (CBP20 and CBP80, possibly involved in cap proximal intron splicing [55]), derive from single-copy genes [56]. These single-copy gene products may work with all pre-mRNAs, including the ones with U12-type introns. Surprisingly, mutation of CBP80 (*ABH1*) is not lethal and is non-pleiotropic. The *abh1 *plants show ABA-hypersensitive closure of stomata and reduced wilting during drought [57].

Many splicing factors have been identified previously in *Arabidopsis*, including two U2AF65, two U2AF35, and 18 SR proteins [58-67]. The U2AF35-related protein atUrp, which could interact with U2AF65 and position RS-domain-containing splicing factors [68], is also present in the *Arabidopsis *genome. Although the *Urp *gene is expressed ubiquitously in human tissues, no ESTs from this gene were found in *Arabidopsis*. Three copies of *PTB/hnRNP-I *genes were identified in *Arabidopsis*. The PTB protein competes for the poly-pyrimidine tract with the U2AF large subunit, thus negatively regulating splicing [69].

We also identified a homolog related to atU2AF^65 ^(At2g33440) and an additional SR protein (At2g46610). The U2AF^65^-related protein (atULrp, At2g33440) has 247 amino acids and shares over 40% similarity with the carboxy terminal region of the two atU2AF^65 ^homologs. Only one RRM can be identified in atULrp, in contrast to three RRMs and one amino-terminal RS domain in atU2AF^65 ^proteins, and there is no apparent RS domain in atULrp. No animal homolog of atULrp could be identified. The function of this one-RRM U2AF^65^-related protein is not clear. As it lacks other functional motifs, it might act as a competitor of U2AF65. A two-RRM U2AF^65 ^protein can be produced through alternative splicing. The 11th intron of atU2AF^65^a can be retained (see RAFL full-length cDNA, gi:19310596) to produce a truncated protein with only the first two RRMs. Interestingly, the last RRM in atU2AF^65^a contains several amino-acid variations from the consensus pattern such that it could not be detected by InterPro and NCBI-CDD searches using default values, also suggesting that perhaps only the first two RRMs are essential.

The additional SR protein belongs to the atRSp31 family and was named atRSp32 (At2g46610). It shares 70% identity and 78% similarity with atRSp31. The protein is 250 amino acids in length and contains two RRMs and some RS dipeptides in the carboxy-terminal region. The gene structure of *atRSp32 *is similar to that of *atRSp31*. Two other genes (*atRSp40 *and *atRSp41*) are in the same family and also have similar exon and intron sizes (see gene structure information at [70]). Similarly to the previous classification of 18 SR proteins [61], the 19 SR proteins (including SR45) can be grouped into four large families of four to five members according to sequence similarity, gene structure and protein domain structure.

The atRSp31 family (atRSp31, atRSp32, atRSp40 and atRSp41) belongs to a novel plant SR family and has no clear animal ortholog. Other families include the SC35 (or SRrp/TASR2) family, SF2/ASF family, and the 9G8 family. *Arabidopsis *has a single copy of the SC35 ortholog and four SC35-like proteins (atSR33, atSCL30a, atSCL30 and atSCL28), which appear to have diverged significantly from SC35. It seems that this divergence predates the split of plants and animals because a similar SC35-like gene family exists in the human genome (SRrp35 and SRrp40). The SRrp35 and SRrp40 were found to antagonize other SR proteins *in vitro *and function in 5' splice-site selection [71]. SF2/ASF has four copies (atSR1/SRp34, atSRp30, atSRp34a and atSRp34b) with similar gene structures and domains. Human 9G8 protein has five homologs in *Arabidopsis*, with three (atRSZp21, atRSZp22 and atRSZp22a) containing one CCHC-type zinc finger and two (atRSZ33, atRSZ32) containing two CCHC-type zinc fingers in addition to an RRM and an RS domain. Interestingly, several SR proteins (atRSZp21, atRSZp22, SR45 and SCL33) were found to interact with atU1-70K, and some SR proteins can interact with each other, thus forming a complicated interaction network to facilitate splice-site selection and spliceosome assembly [3,61-63]. atSR45 was initially regarded as a novel plant SR protein [63], but by virtue of sequence-similarity scores it actually may be the ortholog of the human *RNPS1 *gene, which encodes an EJC protein. Other human SR proteins (SRp20, SRp30c, SRp40, SRp54, SRp55 and SRp75) lack clear orthologs in *Arabidopsis*. We conclude that SR protein families evolved differently in animals and plants from three to four common ancestors, including SC35, SF2/ASF and 9G8/RSZ. The SRrp (SC35-like in plants) family may not be classical SR proteins but they play important roles in splice-site selection.

Proteins in other subgroups, such as 17S U2 snRNP-associated proteins, 35S U5 snRNP-associated proteins, and protein specific to the BΔU1 complex, are also conserved in *Arabidopsis*. The BΔU1 complex is the spliceosome complex captured immediately before catalytic activation. Most proteins in the 35S U5 snRNP are absent in the BΔU1 complex but present in the active B complex, indicating the important roles of 35S U5 snRNP-associated proteins in spliceosome activation [13]. Conservation of these proteins in *Arabidopsis *revealed the same pathway of spliceosome activation in plants. A subcomplex named Prp19 complex in 35S U5 snRNP has a critical role in spliceosome activation [13,72]. All proteins in the human Prp19 complex have homologs in *Arabidopsis*, including a chromosomal duplication pair of *Prp19 *genes and a single copy of the *CDC5 *gene. For the BΔU1 complex, six human genes have homologs, and five of them are single copy in *Arabidopsis*. Two genes (*NPW38BP/SNP70 *and *p220*(*NPAT*)) in the human BΔU1 complex have no apparent *Arabidopsis *homologs.

*Arabidopsis *also lacks an SMN protein complex. In human, the SMN protein (survival of motor neurons) can interact with a series of proteins including Gemin2, Gemin3 (a helicase), Gemin4, Gemin5 and Gemin6 to form an SMN complex, which has important roles in the biogenesis of snRNPs and the assembly of the spliceosome through direct interactions with Sm proteins and snRNA [73]. Although the SMN protein exists in the fission yeast genome (GenBank accession CAA91173), no SMN complex members can be identified in the *Arabidopsis *genome.

### Splicing regulators are expanded in *Arabidopsis*

Splicing regulators are proteins that can either modify splicing factors or compete with splicing factors for their binding site. Important splicing regulators are hnRNP proteins and SR protein kinases. The exact role of phosphorylation of SR proteins in splicing is not yet clear, but SR protein kinases are well conserved and exist as multiple copies in *Arabidopsis*. A total of eight SR protein kinases were identified in *Arabidopsis*, including three Lammer/CLK kinases (AFC1, AFC2 and AFC3), two SRPK1 homologs, and three SPRK2 homologs. The three Lammer/CLK kinases were identified previously, and AFC2 was shown to phosphorylate SR protein *in vitro *[63,74]. Overexpression of tobacco AFC2 homolog PK12 in *Arabidopsis *changed the alternative splice patterns of several genes, including *atSRp30*, *atSR1*/*atSRp34 *and *U1-70K *[75], indicating that these SR proteins may function to modulate splicing in plants.

The heterogeneous nuclear ribonucleoproteins (hnRNPs) bind to splice sites and to binding sites for splicing factors on nascent pre-mRNAs, thus competing with splicing factors to negatively control splicing (reviewed in [76]). Humans have about 20 hnRNP proteins, many of which function in splicing. A total of 35 potential hnRNP proteins possibly related to splicing was found in *Arabidopsis *by sequence-similarity searches, including a superfamily of glycine-rich RNA-binding proteins. This family contains 21 members similar to human hnRNP A1 and hnRNP A2/B1. It can be further divided into two subfamilies. One includes eight proteins containing one RRM, and another has 13 members with two RRMs. 12 of these proteins were identified previously, including AtGRP7, AtGRP8, UBA2a, UBA2b, UBA2c and AtRNPA/B1-6 [11,77,78]. AtGRP7 was found to be able to influence alternative splicing of its own transcripts as well as *AtGRP8 *transcripts [79]. UBA2 proteins can interact with UBP1 and UBA1 proteins, which have three RRMs and one RRM respectively, to recognize U-rich sequences in the 3' untranslated region (UTR) and stabilize mRNA [78]. Although the overexpression of UBA2 did not stimulate splicing of a reporter gene in tobacco protoplasts [78], we cannot rule out the possibility that it could be involved in splicing of other genes.

Other human hnRNPs related to splicing also have homologs in *Arabidopsis*. BLAST searches of the human (CUG)n triplet repeat RNA-binding protein (CUG-BP) against all *Arabidopsis *proteins revealed three putative homologs, including atFCA. atFCA and CUG-BP share similarity within the RRMs and a region approximately 40 amino acids in length. An additional protein (At2g47310) related to FCA was identified and named FCA2, as it shares about 50% similarity with FCA. The FCA proteins have two RRMs and a WW domain, which interact with the FY protein, a homolog of yeast polyadenylation factor Psf2p [80,81]. The FCA-FY complex negatively regulates the FCA protein by favoring a polyadenylation site from the third intron of FCA pre-mRNA [80,82]. FCA may be a multifunctional protein involved in mRNA processing, as human CUG-BP can function in both alternative splicing and deadenylation [83]. We also list 15 previously identified hnRNP-like proteins and two additional homologs as possible splicing regulators. The UBP1 proteins can strongly enhance splicing of some introns in protoplasts [84], whereas UBA1, RBP45 and RBP47 proteins have no similar function [78,85].

### Unclassified splicing protein candidates

In addition to the 260 proteins in the above three categories, there are also 84 *Arabidopsis *proteins corresponding to human spliceosome-associated proteins identified in recent proteomic studies [15-18]. Some of these proteins function in other processes, such as transcription, polyadenylation and even translation. Their association with spliceosomes provides evidence for the coupling of splicing and other processes. Other proteins have no known functions. Only 35.8% of the proteins in this category are duplicated in *Arabidopsis*. We also identified a total of 51 *Arabidopsis *protein-coding genes similar to known splicing proteins. They have conserved domains and some level of sequence similarity to known splicing factors. We did not include these two categories in Table 2, but detailed information about them is available at ASRG [39].

## Distribution and duplication of *Arabidopsis *splicing-related genes

The distribution of *Arabidopsis *snRNA and splicing-related proteins across the genome is shown in Figure 2 and at the ASRG website. Overall, the genes appear evenly distributed on the chromosomes, with several small gene clusters. Only four snRNA genes are located on chromosome 2, three of which are U2 snRNA genes. No U4 snRNA gene is located on chromosome 4. For the protein-coding genes, most functional categories have members located on each chromosome. The only exception is the SR protein kinase family, which has no member on chromosome 1. Interestingly, chromosome 1 contains the most snRNP proteins and splicing factors, but has the fewest splicing regulators. Several gene clusters encoding splicing-related proteins were also identified. Some clusters, such as tandemly duplicated gene pairs, include genes from the same category. One cluster located on chromosome 4 includes four genes encoding tri-snRNP proteins (atTri65a, atTri65b, atTri65c and atTri15.5c, homologs of tri-snRNP 65-KD protein and 15.5 KD protein). Two other clusters, *atU2A-atCdc5 *and *atCUG-BP1-atU1C*, include genes from different functional categories. No clear clusters of genes for snRNA-splicing-related proteins were identified. Although about one third of snRNA genes are located near other protein-coding genes, none of their neighboring genes is related to splicing. As a caveat, we should point out that our snRNA gene determination strongly suggests annotation errors in overlapping protein-coding gene models. Thus, atU2-1, atU2.3, atU4.2, atU4-11p, atU5-13 and atU6.26 overlap gene models At1g16820, At3g57770, At3g06895, At1g68390, At5g53740 and At3g13857, respectively, but none of these models is well supported by cDNA or EST evidence (see displays linked at ASRG [30]).

The 260 proteins in the first three categories could be grouped into 130 families, 66 of which consist of multiple members. The duplication rate is over 50%, which is higher than the 44% duplication rate of *Arabidopsis *transcription factors [86]. As shown in Table 3, about 50% of genes encoding snRNP proteins, 43% of splicing factors, and 78% of splicing regulators have duplications. The much higher duplication rate of splicing regulators may reflect diversification in splicing control.

At least 130 duplication events are required to yield the 260 proteins from 130 families given one single-copy ancestor per family. Thirty-three duplication events (about a quarter of the total) are likely to be the result of chromosome duplications. The chromosomal duplication ratio is 18.9-27.5% among the three groups (see Table 3). Some snRNA genes pairs, such as *U2-14*/*U2-10*, *U5-3*/*U5-5 *and (*U6.1 U6.26*)/(*U6-8p U6-9p*), may also have been produced by chromosome duplication. The C.D.2-3 region (chromosome duplication region between chromosomes 2 and 3, see [87]) has the most splicing-related gene pairs. Six genes in this region on chromosome 2 were duplicated in the same order on chromosome 3. EST evidence shows that all these genes are expressed. Three U5 snRNA genes (*U5.1*, *U5.1b *and *U5-4*) and four U2 snRNA genes (*U2.2*, *U2.3*, *U2.4 *and *U2.6*) also are located in the same region on chromosome 3. No U5 and U2 homologs exist in the corresponding region on chromosome 2, suggesting that the snRNA duplication events in that region may have happened after the chromosome duplication event, or that the snRNA duplicates were lost subsequent to chromosome duplication.

Chromosomal duplication rather than individual gene duplication appears to be the predominant mode of amplification for some types of genes. As shown in Table 2, the 24 genes encoding core proteins have nine duplication pairs, five of which can be attributed to chromosomal duplications. The 19 SR protein genes include eight duplication pairs, six of which are probably the results of chromosomal duplications. At least five chromosomal duplication events contributed to the superfamily of 21 hnRNP glycine-rich RBD and A/B genes. It is not clear why these functional categories have high chromosomal duplication ratios. It is possible that chromosomal duplication could create positive selection to maintain similar copy numbers of other genes encoding proteins that interact with the products of already duplicated genes.

## Alternative splicing of *Arabidopsis *splicing-related genes

According to EST/cDNA alignments, 80 of the 260 protein coding genes show 66 alternative splicing events. This rate (30.8%) is much higher than the overall frequency of alternative splicing in *Arabidopsis*, which is about 13% using the same criteria (2,747 genes out of 20,446 genes with EST/cDNA evidence; B.-B.W. and V.B., unpublished work). As shown in Table 4, the snRNP protein-coding genes have the lowest alternative splicing ratio (24.2%), whereas the ratios for splicing factor and splicing regulator genes are both over 33%. More than half of the genes encoding EJC proteins, proteins specific for the BΔU1 complex, SR proteins, U11/U12 snRNP-specific proteins and U1 snRNP proteins undergo alternative splicing.

Among different types of alternative splicing, intron retention is the most abundant of the alternative transcripts identified for the 260 classified splicing-related genes. As shown in Table 4, 44 of the total 80 alternative splicing genes (about 55%) involve intron retention, 28 (35%) involve alternative acceptor-site selection and 15 (18.7%) are due to exon skipping. Compared with the corresponding ratio in all *Arabidopsis *alternative splicing events (55.3% intron retention, 23.4% alternative acceptor-site selection and 6.3% exon skipping; B.-B.W. and V.B., unpublished work), the ratio of intron retention in splicing-related genes is similar and the ratio of exon skipping is higher. Interestingly, only one of the 20 splicing regulator genes processed by alternative splicing (about 5%) shows exon skipping, indicating that exon skipping is an important post-transcriptional method for controlling the expression of splicing factor coding genes but not the splicing regulator genes.

## Discussion

Previous studies had determined 30 snRNA genes and 46 protein-coding genes related to splicing in *Arabidopsis *(see Tables 1 and 2). In this study, we have computationally identified an additional 44 snRNA genes (Table 1) and 349 protein-coding genes (Table 2) that also may be involved in splicing. Among the five types of U snRNAs, U6 is the most conserved and U1 is the least conserved. We identified seven U1-U4 snRNA gene clusters. We were surprised to see so many U1-U4 clusters in *Arabidopsis*. In *Drosophila*, four snRNA clusters were reported [4], but none of them includes U1-U4 gene pairs. It is likely that a U1-U4 snRNA cluster existed in a progenitor of the current *Arabidopsis *genome, which was duplicated several times to form the extant seven clusters. The non-clustered U1 and U4 snRNA genes may have arisen by individual gene duplication or gene loss in duplicated clusters.

Among the proteins involved in splicing, most animal homologs are conserved in plants, indicating an ancient, monophylytic origin for the splicing mechanism. A striking feature of plant splicing-related genes is their duplication ratio. Fifty percent of the splicing genes are duplicated in *Arabidopsis*. The duplication ratio of the splicing-related genes increases from genes encoding snRNP proteins to genes encoding splicing regulators. These data strongly suggest that the general splicing mechanism is conserved, but that the control of splicing may be more diverse in plants.

The high duplication ratio of *Arabidopsis *splicing-related genes could be the result of evolutionary selection. Unlike animals, which can move around to maintain more homogeneous physiological conditions, plants are exposed to a larger range of stress conditions such as heat and cold. The duplicates will more probably be maintained in the genome as their functions become diversified, and potentially plant-specific, to ensure the fidelity of splicing under such varied conditions. Chromosome duplication has produced several Sm proteins, SR proteins and hnRNP proteins in *Arabidopsis*, which in turn could create positive selective pressures influencing the rate of duplication for functionally related genes. Because chromosome duplication occurred differentially within each plant lineage, we would expect different duplication patterns of these genes in, for example, monocots and dicots.

**Figure 3:**
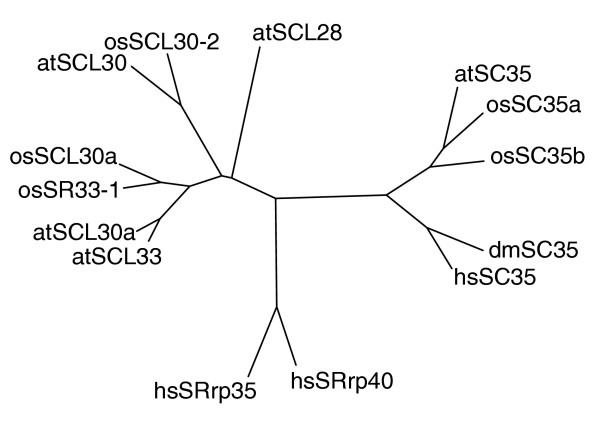
Phylogenetic tree of the SC35 protein family. The phylogenetic tree was constructed on the basis of protein sequence alignments of the SC35 homologs in human, *Drosophila*, *Arabidopsis *and rice. The GenBank accession numbers for the sequences are as follows: hsSC35, Q01130; hsSRrp40, AAL57514; hsSRrp35: AAL57515; dmSC35, AAF53192; atSC35, NP_851261; atSR33/SCL33, NP_564685; atSCL30a, NP_187966; atSCL30, NP_567021; atSCL28, NP_197382; osSC35a, BAC79909; osSC35b, BAD09319; osSR33-1, AAP46199; osSCL30a/SR33-2, BAC799901; osSCL30-2, BAD19168. The sequences were aligned using CLUSTALW [22] with default parameters, and the phylogenetic tree was produced according to the neighbor-joining method using PAM substitution model distances as implemented in the PHYLIP package [103].

To confirm the above hypothesis, we searched the recently sequenced rice genome using the five *Arabidopsis *SC35 and SC35-like proteins as probes. Eight distinct genome loci were found to encode SC35 and SC35-like proteins, including three homologs of atSC35, two homologs of atSR33/SCL33 and atSCL30a, two homologs of atSCL30, and one homolog of atSCL28. Five of the eight rice genes are currently annotated in GenBank with accession numbers BAC79909 (osSC35a), BAD09319 (osSC35b), AAP46199 (osSR33-1), BAC799901 (osSCL30a/osSR33-2), and BAD19168 (osSCL30-1). As shown in the phylogenetic tree displayed in Figure 3, the two rice SC35 genes and atSC35 are likely to be orthologs of the animal SC35 gene. The other sequences cluster in SC35-like (SRrp/TASR) clades, indicating that the SC35 and SRrp/TASR genes diverged before the divergence of monocot and dicot plants (the divergence presumably happened even before the divergence of animals and plants, as described earlier). In addition, there are species-specific duplications. Thus, the *Arabidopsis *chromosomal duplication pair atSR33 - atSCL30a forms a clade, while their rice copies (osSR33-1 and osSCL30a) form another clade. Also there are additional duplications for the rice SC35 and SCL30 genes. We are currently working to identify all rice splicing related genes. The complete sets of these genes in two plant species should provide a good foundation for assessing similarities and differences in splicing mechanisms used by monocot and dicot plants.

As introns evolve rapidly, the mechanism to recognize and splice them should either evolve correspondingly or be flexible enough to accommodate the changes. It seems that plants deploy the most economic and practical way by keeping a largely conserved splicing mechanism and a very flexible recognition and control mechanism. Direct evidence comes from the presence of plant-specific splicing proteins, such as the novel SR protein family and the superfamily of hnRNP A/B. The absence of SMN complex and some yeast U1 snRNP proteins in *Arabidopsis *indicates that other organisms also have integrated new proteins or pathways into the splicing mechanism over the course of evolution relative to other eukaryotes. Other evidence supporting the conserved splicing but flexible regulating mechanism include differential conservation among U snRNAs (U1 snRNAs are less conserved than U6 snRNAs) and high alternative splicing frequency in U1 snRNP proteins, SR proteins and hnRNP proteins. The SR proteins and U1 snRNP proteins are involved in early steps of splicing and 5' and 3' splice-site selection; multiple isoforms of these proteins may be functionally significant in the control of splicing.

It is interesting to note that the overall alternative splicing frequency in splicing related genes is much higher than the frequency averaged over all *Arabidopsis *genes. More than half of SR proteins and U1 snRNP proteins show alternative splicing. Alternative splicing might increase protein diversity derived from splicing-related genes, which would further add flexibility to the splicing mechanism. The high frequency of alternative transcripts from splicing related genes raises another interesting question - how is splicing regulated in these splicing-related genes? One possible answer is that some splicing-related genes may be autoregulated. Accumulation of one transcript would feed back to inhibit/promote other isoforms. Several splicing-related genes have been reported to be regulated in this way. For example, AtGRP7 (hnRNP A/B superfamily) is a circadian clock-regulated protein which negatively autoregulates its expression [79]. When the AtGRP7 protein accumulates over the circadian cycle, it promotes production of alternative transcripts which use a cryptic 5' splice site. As a result of message instability, the alternative transcripts contain pre-mature stop codons and do not accumulate to high levels, thus decreasing the level of AtGRP7 protein [79]. atSRp30 has similar effects on its own transcripts [65]. Another possible answer is that some splicing-related genes might regulate the splicing of other splicing-related genes. For example, overexpression of *AtGRP7 *and *atSRp30 *is known to affect the splicing of *AtGRP8 *and *atSR1*, respectively [65,79]. A third possibility is that the environment could affect the alternative splicing pattern. A good example is the *SR1 *gene. The ratio of two transcripts from the *SR1 *gene (SR1B/SR1) increases in a temperature-dependent manner [67]. Generally, heat or cold stress could cause intron retention in some splicing regulators, which could further alter the splicing pattern of other genes. The fourth possible regulators are intronless genes. Combining all these possibilities, a pathway to regulate splicing could be inferred as follows: environmental changes → splicing pattern changes in some specific splicing-related genes and/or intronless genes → expression pattern changes (including splicing pattern changes) in general splicing related genes → changes in splicing patterns for specific genes.

## Conclusions

A large number of *Arabidopsis *splicing-related genes were computationally identified in this study by means of sequence comparisons and motif searches, including a tentative *U4atac *snRNA gene containing all conserved motifs, a new SR protein-coding gene (*atRSp32*) belonging to the atRSp31 family, and several genes related to genes encoding known splicing-related proteins (atULrp and atFCA2). A web-accessible database containing all the *Arabidopsis *splicing related genes has been constructed and will be expanded to other organisms in the near future. This compilation should provide a good foundation to study the splicing process in more detail and to determine to what extent these genes are conserved across the entire plant kingdom. Our data show that about 50% of the splicing-related genes are duplicated in *Arabidopsis*. The duplication ratios for splicing regulators are even higher, indicating that the splicing mechanism is generally conserved among plants, but that the regulation of splicing may be more variable and flexible, thus enabling plants to respond to their specific environments.

## Materials and methods

### Search for *Arabidopsis *snRNAs

Sequences of the 15 experimentally identified major snRNAs were downloaded from GenBank. The two minor snRNAs sequences were compiled from the literature [28]. These genes were used to search against the *Arabidopsis *genome at the AtGDB BLAST server [88] and at the SALK T-DNA Express web server [89]. Our initial analysis was based on Release 3.0 of the *Arabidopsis *genome (GenBank accession numbers NC_003070.4, NC_003071.3, NC_003074.4, NC_003075.3, and NC_003076.4). Local BLAST [21] was used to derive the locations of the snRNA homologs from more recently sequenced regions of the genome. Criteria used for local BLAST were 'e 1 -F F -W 7' (cutoff eval is 1, dust filter on, with a minimum word size of 7). Human and maize snRNAs were also included as query sequences, and all hits with e-values less than 10^-5 ^were regarded as possible homologs. A total of 70 major snRNAs and three minor snRNAs were identified by this method. Each major snRNA type has 10-18 copies in the genome. A tentative gene name and gene model were assigned to each snRNA gene after comparison with the snRNAs identified in MATDB [90]. Sequence-similarity values were based on BLAST alignments.

### Search for *Arabidopsis *splicing-related proteins

A three-round BLAST search strategy was used to identify *Arabidopsis *splicing related protein-coding genes. First, sequences of splicing-related proteins from human and *Drosophila *were downloaded from GenBank according to several recent proteomic studies [15-18] and the website compilation of Stephen Mount's group available at [91]. Human hnRNP proteins identified in a recent review [76] were downloaded from GenBank. All these sequences were used as queries in a local BLAST search against *Arabidopsis *annotated proteins (obtained from TIGR at [92]). All hits with an e-value less than 10^-10 ^were collected as candidates. Many of these candidates had highly significant e-values (usually 10^-30 ^or below and much lower than other hits). These candidates were regarded as true homologs.

In the second step, all identified true homologs were used to query the *Arabidopsis *protein set again. An e-value of 10^-20 ^was used as a cutoff value to find possible paralogs of the true homologs. Sequences identified in both rounds of BLAST hits were regarded as main candidates for splicing related proteins.

Finally, the main candidates were queried against GenPept and all annotated human proteins (obtained from Ensembl [93]). All candidates with significant similarity to proteins unrelated to splicing were removed from the main candidate list, and all candidates with significant similarity to proteins related to splicing were regarded as true splicing-related genes and were promoted to the status of true homologs. The remaining candidates were regarded as unclassified splicing-related proteins. BLAST results were initially analyzed by MuSeqBox [94]). Two custom scripts were written to read MuSeqBox output files, largely automating the search procedure.

### Gene structure and chromosomal locations

The gene structure and chromosomal locations for the genes encoding splicing-related proteins were retrieved from AtGDB [95]. The chromosomal locations of the snRNA genes were inferred from the BLAST results. The location maps (Figure 1) were generated using the AtGDB advanced search function [96]. Spliced alignments of ESTs and cDNAs generated by GeneSeqer [97] were used to verify gene models. Gene structure information was used as an important criteria to group homologs into gene families.

### Protein domains

InterProScan 3.3 was downloaded from [98] and was subsequently used to search protein domain databases using default parameters [99]. A Perl script was written to process the text results from InterProScan. Protein domain information was used in comparisons of homologs from different species. The search of the National Center for Biotechnology Information Conserved Domain Database (NCBI-CDD) [100] was conducted manually for certain genes to confirm the InterPro results.

### Duplication source

The gene families with multiple copies were inspected to determine whether they were likely to have derived from chromosome-duplication events. Gene models of the duplicated gene were searched against the gene list of each chromosome redundancy region at MATDB [101]. If the gene and its duplicate were both in the list, they were regarded as a chromosome duplication pair. Otherwise, they were assumed to be produced by random gene duplication.

### Identification of alternative splicing

All *Arabidopsis *ESTs and cDNAs were aligned against the genome using the spliced alignment program GeneSeqer as made available through AtGDB [102]. We retrieved the intron and exon coordinates of the reliable cognate alignments from the database. Scripts were written to identify introns that overlap with other introns or exons. We defined the alternative splicing cases as follows: alternative donor (AltD): an intron has the same 3'-end coordinate but different 5'-end coordinate as another overlapping intron; alternative acceptor (AltA): an intron has the same 5'-end coordinate but different 3'-end coordinate as another intron; alternative position (AltP): an intron has different 5'-end and 3'-end coordinates as another overlapping intron; exon skipping (ExonS): an annotated intron completely contains an alternatively identified exon in the same transcription direction; intron retention (IntronR): an annotated intron is completely contained by an alternatively identified exon.

### Database and interface construction

Details about each splicing-related gene were saved in a MySQL database. PHP scripts were written to interact with the database and generate the interface web pages. Text and BLAST searches were implemented by Perl-cgi scripts.

## Figures and Tables

**Table 1:** *Arabidopsis *snRNA genes

Gene	GeneID	Chromosome	Strand	From	To	Length (nucleotides)	e-value	Similarity	GenBank ID
atU1a*	At5g49054	5	-	19903323	19903158	166	1E-89	1-166, 100%	gi17660
*atU1-2*	At4g23415	4	+	12225621	12225786	166	1E-58	1-166, 92%	gi22293582
*atU1-3*	At5g51675	5	+	21013986	21014149	164	4E-55	3-166, 91%	
*atU1-4*	At5g25774	5	-	8972971	8972807	165	2E-51	1-166, 90%	gi22293583
*atU1-5*	At1g08115	1	-	2538238	2538073	166	1E-46	1-166, 89%	gi22293581
*atU1-6*	At3g05695	3	+	1681815	1681977	163	4E-40	4-166, 87%	
*atU1-7*	At3g05672	3	+	1657766	1657928	163	4E-40	4-166, 87%	gi22293580
*atU1-8*	At5g27764	5	+	9832576	9832740	165	1E-39	1-166, 87%	
*atU1-9*	At5g26694	5	-	9494594	9494430	165	1E-27	1-166, 84%	
*atU1-10*	At1g11884	1	-	4007396	4007236	161	1E-18	4-61, 93%; 80-166, 88%	
*atU1-11p*	At4g16645	4	+	9370786	9370841	56	7E-17	4-59, 94%	
*atU1-12p*	At4g23565	4	-	12298871	12298802	70	1E-15	94-163, 90%	
*atU1-13p*	At5g49524	5	-	20112431	20112275	157	2E-14	4-50, 91%; 91-166, 88%	
*atU1-14p*	At1g35354	1	+	12986822	12986908	87	1E-06	10-60, 88%; 84-118, 88%	
*atU2-1*	At1g16825	1	+	5758381	5758575	195	2E-88	1-196, 96%	
atU2.2*	At3g57645	3	+	21357718	21357913	196	1E-107	1-196, 100%	gi17661
*atU2.3*	At3g57765	3	-	21408595	21408400	196	1E-95	1-196, 97%	gi17662
*atU2.4*	At3g56825	3	-	21052994	21052800	195	5E-86	1-196, 95%	gi17663
*atU2.5*	At5g09585	5	+	2975013	2975208	196	7E-79	1-196, 93%	gi17664
*atU2.6*	At3g56705	3	+	21015472	21015667	196	1E-83	1-196, 94%	gi17665
*atU2.7*	At5g61455	5	-	24730829	24730634	196	5E-86	1-196, 95%	gi17666
*atU2-8*	At5g67555	5	+	26966884	26967079	196	5E-86	1-196, 95%	
*atU2.9*	At4g01885	4	+	815273	815466	194	2E-82	1-194, 94%	gi17667
*atU2-10*	At2g02938	2	+	849777	849972	196	3E-93	1-196, 96%	gi22293586
*atU2-10b/12*	At2g02940	2	+	852859	853054	196	3E-93	1-196, 96%	
*atU2-11*	At1g09805/09895	1	-	3180736	3180547	190	8E-85	1-190, 95%	
*atU2-13*	At2g20405	2	+	8809169	8809364	196	3E-81	1-196, 94%	gi22293584
*atU2-14*	At1g14165	1	+	4842274	4842469	196	3E-81	1-196, 94%	gi22293585
*atU2-15*	At5g62415	5	+	25083790	25083985	196	4E-74	1-196, 92%	
*atU2-16*	At5g57835	5	-	23448717	23448522	196	2E-67	1-196, 92%	
*atU2-17*	At5g14545	5	-	4690105	4690008	98	3E-44	1-98, 97%	
*atU2-18p*	At3g26815	3	+	9881236	9881303	68	2E-14	1-68, 89%	
atU4.1*	At5g49056	5	-	19902970	19902817	154	4E-80	1-154, 99%	gi17673
*atU4.2*	At3g06900	3	-	2178343	2178190	154	2E-75	1-154, 98%	gi17674
*atU4.3p*	At5g49526	5	-	20112072	20112030	43	2E-11	15-57, 95%	gi17675
*atU4-4*	At1g49242/49235	1	-	18222354	18222201	154	2E-75	1-154, 98%	gi22293588
*atU4-5*	At5g25776	5	-	8972618	8972465	154	1E-70	1-154, 96%	
*atU4-6*	At1g11886	1	-	4007020	4006867	154	1E-70	1-154, 96%	gi22293587
*atU4-7*	At5g27766	5	+	9832934	9833083	150	7E-66	1-150, 96%	
*atU4-8*	At5g26996	5	-	9494230	9494081	150	7E-66	1-150, 96%	
*atU4-9*	At1g79965	1	+	30086031	30086168	138	9E-47	18-154, 92%	
*atU4-10*	At1g35356	1	+	12987189	12987313	125	3E-34	1-124, 90%	
*atU4-11p*	At1g68395	1	+	25647322	25647396	75	9E-07	18-37, 100%; 60-102, 90%	
atU5.1*	At3g55865	3	-	20740607	20740503	105	6E-35	1-105, 94%	gi17676
*atU5.1b*	At3g55855	3	-	20736881	20736780	102	7E-38	1-102, 96%	gi22293592
*atU5-2*	At1g65115	1	+	24194482	24194586	105	1E-39	1-105, 96%	
*atU5-3*	At1g70185	1	+	26433396	26433497	102	7E-38	1-102, 96%	gi22293590
*atU5-4*	At3g55645	3	+	20653843	20653947	105	3E-37	1-105, 95%	
*atU5-5*	At1g24105/24095	1	-	8525204	8525103	102	2E-35	1-102, 95%	gi22293591
*atU5-6*	At1g04475	1	-	1215831	1215730	102	2E-35	1-102, 95%	gi22293589
*atU5-7*	At4g02535	4	-	1114629	1114528	102	1E-30	2-103, 93%	
*atU5-8*	At3g25445	3	-	9227212	9227116	97	1E-20	5-101, 89%	
*atU5-9*	At1g79545	1	-	29928543	29928447	97	1E-20	5-101, 89%	
*atU5-10*	At5g14547	5	-	4690412	4690370	43	3E-12	24-67, 97%	
*atU5-11*	At5g54065	5	-	21957066	21957023	44	2E-10	20-64, 95%	
*atU5-12*	At1g71355	1	+	26895255	26895298	44	2E-10	20-64, 95%	
*atU5-13*	At5g53745	5	-	21829988	21829943	46	3E-09	24-70, 93%	
atU6.1*	At3g14735	3	+	4951596	4951697	102	1E-51	1-102, 100%	gi16516
*atU6.26*	At3g13855	3	+	4561111	4561212	102	2E-49	1-102, 99%	gi16517
*atU6.29*	At5g46315	5	+	18804616	18804717	102	2E-49	1-102, 99%	gi16518
*atU6-2*	At5g62995	5	+	25296825	25296926	102	1E-51	1-102, 100%	
*atU6-3*	At4g27595	4	+	13782215	13782316	102	1E-51	1-102, 100%	
*atU6-4*	At4g03375	4	-	1483121	1483020	102	1E-51	1-102, 100%	
*atU6-5*	At4g33085	4	-	15965258	15965158	101	8E-37	1-101, 94%	
*atU6-6*	At4g35225	4	+	16754836	16754931	96	1E-32	1-102, 93%	
*atU6-7*	At2g15532	2	+	6784793	6784869	77	7E-25	4-80, 93%	
*atU6-8p*	At1g52605	1	+	19596398	19596476	96	2E-19	4-99, 87%	
*atU6-9p*	At1g53465	1	-	19960538	19960485	54	9E-09	21-74, 88%	
*atU6-10p*	At3g45705	3	+	16792802	16792888	87	2E-06	1-46, 89%; 62-100, 89%	
*atU6-11p*	At5g11085	5	-	3522167	3522143	25	9E-06	1-25, 100%	
atU12*	At1g61275	1	+	22606785	22606960	176	1E-95	1-176, 100%	^†^gi22293600
atU6atac*	At5g40395	5	-	16183534	16183413	122	1E-63	1-122, 100%	^†^
*atU6atac-2*	At1g21395	1	-	7491489	7491378	112	5E-20	1-65, 95%; 81-110, 93%	
*atU4atac*	At4g16065	4	+	9096374	9096532	159	N/A	N/A	

Chromosomal locations were determined by conducting BLAST searches against the *Arabidopsis *genome (Release 5.0). *The gene used for query in the BLAST search; ^†^atU12 and atU6atac sequences, which were experimentally identified [28]. Their sequences were compiled manually from the cited paper. The GenBank gi numbers for the chromosome sequences used are as follows: chromosome 1, 42592260; chromosome 2, 30698031; chromosome 3, 30698537; chromosome 4, 30698542; chromosome 5, 30698605.

**Table 2:** *Arabidopsis *splicing-related proteins

Human homologs	*Saccharomyces cerevisiae*	Gene name	GeneID	Chromosome	Tnb	AltS	Chromosomal duplication	Protein domain	Reference
**1.1 Sm core proteins**									
SmB	SmB1	*atSmB-a*	At5g44500	5	7		>4-5a	Sm, 1	
		*atSmB-b*	At4g20440	4	21	IntronR (1);	>4-5a	Sm, 1	
		
SmD1	SmD1	*atSmD1-a*	At3g07590	3	7	IntronR (1);		Sm, 1	
		*atSmD1-b*	At4g02840	4	13			Sm, 1	
		
SmD2	SmD2	*atSmD2-a*	At2g47640	2	7	AltA (1); AltD (1);		Sm, 1	
		*atSmD2-b*	At3g62840	3	25	AltA (1);		Sm, 1	
		
SmD3	SmD3	*atSmD3-a*	At1g76300	1	9		>1-1c	Sm, 1	
		*atSmD3-b*	At1g20580	1	7		>1-1c	Sm, 1	
		
SmE	SmE	*atSmE-a*	At4g30330	4	2		>2-4b	Sm, 1	
		*atSmE-b*	At2g18740	2	10	AltA (1);	>2-4b	Sm, 1	
		
SmF	SmF	*atSmF*	At4g30220	4	6			Sm, 1	
		
SmG	SmG	*atSmG-a*	At2g23930	2	13			Sm, 1	
		*atSmG-b*	At3g11500	3	9			Sm, 1	
		
LSM2	LSm2	*atLSM2*	At1g03330	1	7			Sm, 1	
		
LSM3	LSm3	*atLSM3a*	At1g21190	1	6		>1-1c	Sm, 1	
		*atLSM3b*	At1g76860	1	16		>1-1c	Sm, 1	
		
LSM4	LSm4	*atLSM4*	At5g27720	5	13			Sm, 1	
		
LSM5	LSm5	*atLSM5 /SAD1*	At5g48870	5	7	AltA (1);		Sm, 1	[47]
		
LSM6	LSm6	*atLSM6a*	At3g59810	3	7		>2-3	Sm, 1	
		*atLSM6b*	At2g43810	2	5		>2-3	Sm, 1	
		
LSM7	LSm7	*atLSM7*	At2g03870	2	6			Sm, 1	
LSM8	LSm8	*atLSM8*	At1g65700	1	9			Sm, 1	
		
LSM1	LSm1	*atLSM1a*	At1g19120	1	8			Sm, 1	
		*atLSM1b*	At3g14080	3	9	IntronR (1);		Sm, 1	

									
**1.2 U1 snRNP specific proteins**									
U1A Subunit	Mud1	*atU1A*	At2g47580	2	14	ExonS (1);		RRM, 2	[49]
		
U1C Subunit	Yhc1	*atU1C*	At4g03120	4	5			C2H2, 1; mrCtermi, 3	
		
U1-70K	Snp1	*atU1-70K*	At3g50670	3	32	IntronR (1);		RRM, 1	[48]
		
-	Prp39	*atPrp39a*	At1g04080	1	12	ExonS (6);		HAT, 7; TPR-like, 1	
		*atPrp39b*	At5g46400	5	1			HAT, 4;	
		
FBP11	Prp40	*atPrp40a*	At1g44910	1	10	IntronR (1);		WW, 2; FF, 5	
FBP11	Prp40	*atPrp40b*	At3g19670	3	5			WW, 2; FF, 5	
		
Luc7-like protein	Luc7	*atLuc7a*	At3g03340	3	6			DUF259, 1	
		*atLuc7b*	At5g17440	5	8			DUF259, 1	
		
Related to Luc7-like protein	Luc7	*atLuc7-rl*	At5g51410	5	7	IntronR (1);		DUF259, 1	

									
**1.3 17S U2 snRNP specific proteins**									
U2A' Subunit	Lea1p	*atU2A*	At1g09760	1	21			LRR 4;	
		
U2B" Subunit	Msl1p	*atU2B"a*	At1g06960	1	6	AltD (1);	>1-2a	RRM, 2	
		*atU2B"b*	At2g30260	2	13	AltA (1); IntronR (1);	>1-2a	RRM, 2;	
		
SF3a120/SAP114 Subunit	Prp21p	*atSAP114-1a*	At1g14650	1	17	AltB (1);		SWAP/Surp, 2; Ubiquitin, 1	
		*atSAP114-1b*	At1g14640	1				SWAP/Surp, 2	
		*atSAP114-2*	At5g06520	5				SWAP/Surp, 4	
		*atSAP114-3*	At4g16200	4	1			SWAP/Surp, 3	
		*atSAP114p*	At4g15580	4				SWAP/Surp, 3; Ubiquitin, 1	
		
SF3a60/SAP61 Subunit	Prp9p	*atSAP61*	At5g06160	5	10	AltD (1);		C2H2, 1	
		
SF3a66/SAP62 Subunit	Prp11p	*atSAP62*	At2g32600	2	13			C2H2, 1;	
		
SF3b120/SAP130 Subunit	Rse1p	*atSAP130a*	At3g55200	3	6			CPSF_A, 1; WD40-like, 1	[50]
		*atSAP130b*	At3g55220	3	7			CPSF_A, 1; WD40-like, 1	[50]
		
SF3b150/SAP145 Subunit	Cus1p	*atSF3b150*	At4g21660	4	16			PSP, 1; DUF382, 1	
		*atSF3b150p*	At1g11520	1					
		
SF3b160/SAP155 Subunit	Hsh155	*atSAP155*	At5g64270	5	11			HEAT, 1; ARM, 2; SAP_155, 1	
		
SF3b53/SAP49 Subunit	Hsh49p	*atSAP49a*	At2g18510	2	20			RRM, 2	
		*atSAP49b*	At2g14550	2				RRM, 2	
		
p14	Snu17p	*atP14-1*	At5g12190	5	7			RRM, 1;	
		*atP14-2*	At2g14870	2				RRM, 1;	
		
SF3b 14b /PHP5A	Rds3p	*atSF3b_14b-a*	At1g07170	1	10		>1-2a	UPF0123, 1;	
		*atSF3b_14b -b*	At2g30000	2	8		>1-2a	UPF0123, 1;	
		
SF3b 10		*SF3b10a*	At4g14342	4	11			SF3b10, 1;	
		*SF3b10b*	At3g23325	3	6			SF3b10, 1;	

									
**1.4 U5 snRNP specific proteins**									
15 kD Subunit	Dib1p	*atU5-15*	At5g08290	5	28			DIM1, 1; Thioredoxin_2; 1	
		
40 kD Subunit		*atU5-40*	At2g43770	2	21			WD-40, 7;	
		
100 kD Subunit	Prp28p	*atU5-100KD*	At2g33730	2	13			DEAD, 1; Helicase_C, 1	
		
102 KD/Prp6-like	Prp6p	*atU5-102KD*	At4g03430	4	18			Ubiquitin, 1; TPR, 3; HAT, 15; TPR-like, 2; Prp1_N, 1	
		
116 kD Subunit /elongation	Snu114p	*atU5-116-1a*	At1g06220	1	19	ExonS (1);		EFG_C, 1; GTP_EFTU, 1; GTP_EFTU_D2; 1; Small_GTP, 1; EFG_IV, 1;	
		*atU5-116-1b*	At5g25230	5				EFG_C, 1; GTP_EFTU, 1; GTP_EFTU_D2; 1; EFG_IV, 1;	
		*atU5-116-2*	At1g56070	1	214			EFG_C, 1; GTP_EFTU, 1; GTP_EFTU_D2; 1; EFG_IV, 1;	
		*atU5-116-3*	At3g22980	3	3			EFG_C, 1; GTP_EFTU, 1; Small_GTP, 1;	
		
200 kD Subunit/Helicase	Brr2p	*atU5-200-1*	At5g61140	5	11	IntronR (1);		DEAD, 2; Helicase_C, 2; Sec63, 2; ARM, 1	
		*atU5-200-2a*	At1g20960	1	23			DEAD, 2; Helicase_C, 2; Sec63, 2	
		*atU5-200-2b*	At2g42270	2	5			DEAD, 2; Helicase_C, 2; Sec63, 2	
		*atU5-200-3*	At3g27730	3				DEAD, 1; Sec63, 1; RuvA domain 2-like, 1	
		
220 kD Subunit	Prp8p	*atU5-220/Prp8a*	At1g80070	1	33			Mov34, 1	
		*atU5-220/Prp8b*	At4g38780	4	2			Mov34, 1	

									
**1.5 U4/U6 snRNP specific proteins**									
U4/U6-90K / SAP90	Prp3p	*atSAP90-1*	At1g28060	1	10				
		*atSAP90-2*	At3g55930	3					
		*atSAP90-3*	At3g56790	3					
		
U4/U6-60K / SAP60	Prp4p	*atSAP60*	At2g41500	2	8			WD-40, 7; SFM, 1; WD40-like, 1	
		
U4/U6-20K / CYP20		*atTri-20*	At2g38730	2	11			Pro_isomerase, 1	
		
U4/U6-61KD	Prp31	*atU5-61/Prp31a*	At1g60170	1	26			Nop, 1	
		*atU5-61/Prp31b*	At3g60610	3				Nop, 1	
		
U4/U6-15.5K	Snu13p	*atU4/U6-15.5a*	At5g20160	5	18	IntronR (2);		Ribosomal_L7Ae, 1	
		*atU4/U6-15.5b*	At4g12600	4	14			Ribosomal_L7Ae, 1	
		*atU4/U6-15.5c*	At4g22380	4	9			Ribosomal_L7Ae, 1	

									
**1.6 Tri-snRNP specific proteins**									
Tri-65 KD	Snu66p	*atTri65a*	At4g22350	4	7			UCH; 1; ZnF_UBP, 1	
		*atTri65b*	At4g22290	4	20			UCH; 1; ZnF_UBP, 1; Pentaxin, 1	
		*atTri65c*	At4g22410	4				UCH; 1; ZnF_UBP, 1	
		
Tri-110 KD	SAD1	*atTri110*	At5g16780	5	7			SART-1, 1	
		
Tri-27 kD/RY1		*atTri-27 kD/RY1*	At5g57370	5	14				
		
hSnu23/FLJ31121	Snu23p	*atSnu23*	At3g05760	3	7			ZnF_U1, 1;	

									
**1.7 18S U11/U2 snRNP specific proteins**									
U11/U12-35K		*atU11/U12-35kD*	At2g43370	2	7	IntronR (1);		RRM, 1	
		
U11/U12-25K (-99 protein)		*atU11/U12-25K*	At3g07860	3	6	IntronR (2);		C2H2, 1;	
		
U11/U12-65K		*atU11/U12-65K*	At1g09230	1	15	AltA (1);		RRM, 2;PHOSPHOPANTETHEINE, 2;	
		
U11/U12-31K (MADP1)		*atU11/U12-31K*	At3g10400	3	5			RRM, 1;CCHC, 1;	

									
**2.1 Splice site selection**									
U2AF35		*atU2AF35a/AUSa*	At1g27650	1	26			RRM, 1; CCCH, 2;	
		*atU2AF35/AUSb*	At5g42820	5	8			RRM, 1; CCCH, 2;	[58]
		
U2AF65	Mud2	*atU2AF65b/AULa*	At1g60900	1	10			RRM, 3;	[58]
		*atU2AF65a/AULb*	At4g36690	4	29	AltA (1); IntronR (2);		RRM, 2;	[58]
		*atULrp*	At2g33440	2	2			RRM, 1	
		*AUL3p*	At1g60830	1					
		
U2AF35 related protein		*atUrp*	At1g10320	1				RRM, 1; CCCH, 2;	
		
SF1/BBP		*atSF1/BBP*	At5g51300	5	23	IntronR (1);		RRM, 1; CCHC, 2; KH, 1;	
		
CBP20	Cbc1	*atCBP20*	At5g44200	5	8			RRM, 1	[56]
		
CBP80	Cbc2p	*atCBP80*	At2g13540	2	21			MIF4G, 1; ARM, 3	[56]
		
PTB/hnRNP I		*atPTB1*	At1g43190	1	26			RRM, 4;	
		*atPTB2a*	At3g01150	3	21	AltD (1); ExonS (1);	RRM, 2		
		*atPTB2b*	At5g53180	5	17	ExonS (1);		RRM, 2	

									
**2.2 SR proteins**									
SC35		*atSC35*	At5g64200	5	32	AltD (1);		RRM, 1;	[61]
		
SRrp40/TASR-2		*atSR33/atSCL33*	At1g55310	1	12	IntronR (1);	>1-3b	RRM, 1	[63]
		*atSCL30a*	At3g13570	3	32	ExonS (2); IntronR (4);	>1-3b	RRM, 1	[61]
		*atSCL30*	At3g55460	3	14	ExonS (1);		RRM, 1	[61]
		*atSCL28*	At5g18810	5	5			RRM, 1	[61]
		
SF2/ASF		*atSR1/atSRp34*	At1g02840	1	37	AltA (1); IntronR (1);	>1-4	RRM, 2	[64,67]
		*atSRp34a*	At4g02430	4	13	AltA (1); ExonS (1); IntronR (4);	>1-4	RRM, 2	
		*atSRp34b*	At3g49430	3	3	ExonS (1); IntronR (1);	RRM, 2		
		*atSRp30*	At1g09140	1	15	AltA (1);		RRM, 2	[65]
		
9G8		*atRSZp22/atSRZ22*	At4g31580	4	26		>2-4e	RRM, 1; CCHC, 1	[63,66]
		*atRSZp22a*	At2g24590	2	7		>2-4e	RRM, 1; CCHC, 1	[63,66]
		*atRSzp21/atSRZ21*	At1g23860	1	18			RRM, 1; CCHC, 1	[63,66]
		*atRSZ33*	At2g37340	2	30	IntronR (1);	>2-3	RRM, 1; CCHC, 2	[61]
		*atRSZ34*	At3g53500	3	36	AltA (1); IntronR (3);	>2-3	RRM, 1; CCHC, 2	[61]
		
-		*atRSp32*	At2g46610	2	23	AltD (1); IntronR (1);	>2-3	RRM, 2	
		*atRSp31*	At3g61860	3	17	AltA (1);	>2-3	RRM, 2	[59]
		*atRSp41*	At5g52040	5	34	AltA (1);	>4-5b	RRM, 2	[59]
		*atRSp40/atRSP35*	At4g25500	4	15	ExonS (1); IntronR (1);	>4-5b	RRM, 2	[59]

									
**2.3 17S U2 associated proteins**									
hPrp43	Prp43p	*atPrp43-1*	At5g14900	5				HA2, 1	
		*atPrp43-2a*	At3g62310	3	17	AltA (1);	>2-3	DEAD, 1; Helicase_C, 1; HA2, 1	
		*atPrp43-2b*	At2g47250	2	14		>2-3	DEAD, 1; Helicase_C, 1; HA2, 1	
		
SR140		*atSR140-1*	At5g25060	5	11			Surp, 1;RRM, 1;, 1;RPR, 1;	
		*atSR140-2*	At5g10800	5	2			Surp, 1;RRM, 1;RPR, 1;	
		
SPF45		*atSPF45*	At1g30480	1	9			D111/G-patch domain, 1; RRM, 1;	
		
SPF30		*atSPF30*	At2g02570	2	9	AltA (1);		Tudor, 1;	

									
**2.4 35S U5 associated proteins**									
hPrp19*	Prp19p	*atPrp19a*	At1g04510	1	18		>1-2a	WD-40, 7; Ubox, 1;	
		*atPrp19b*	At2g33340	2	27	IntronR (1);	>1-2a	WD-40, 7; Ubox, 1;	
		
CDC5*	Cef1	*atCDC5*	At1g09770	1	12			SANT, 2;	[104]
		
PRL1*	Prp46p	*atPRL1*	At4g15900	4	14			WD-40, 2;WD40like, 1;	
		*atPRL2*	At3g16650	3	6			WD-40, 2;WD40like, 1;	
		
AD-002*	Cwc15p	*atAD-002*	At3g13200	3	22			Cwf_Cwc_15, 1;	
		
HSP73/HSPA8*		*HSP73-1*	At3g12580	3	35			Hsp70, 1;	
		*HSP73-2*	At5g42020	5	51	IntronR (1);		Hsp70, 1;	
		*HSP73-3*	At5g02500	5	553	IntronR (1);		Hsp70, 1;	
		
SPF27/BCAS2*		*atSPF27*	At3g18165	3	15			BCAS2, 1;	
		
beta catenin-like 1*		*atCTNNNBL1*	At3g02710	3	12			Armadillo, 1;ARM, 1;	
		
hSyf1	Syf1p	*atSyf1*	At5g28740	5	7			TPR, 1;HAT, 10;TPRlike, 3;	
		
hSyf3/CRN	Syf3	*atCRN1a*	At5g45990	5				TPR, 1; HAT, 14; TPR-like, 2	
		*atCRN1b*	At3g13210	3				TPR, 1; HAT, 12; TPR-like, 2	
		*atCRN1c*	At5g41770	5	13			TPR, 1; HAT, 14; TPR-like, 2	
		*atCRN2*	At3g51110	3	8			TPR, 1; HAT, 9; TPR-like, 1	
		
hIsy1	Isy1p	*atlsy1*	At3g18790	3	10			Isy1, 1;	
		
GCIP p29	Syf2	*atGCIPp29*	At2g16860	2	12				
		
SKIP	Prp45p	*atSKIP*	At1g77180	1	28			SKIP/SNW, 1;	
		
hECM2	Ecm2p	*atECM2-1a*	At1g07360	1	21		>1-2a	RRM, 1;CCCH, 1;	
		*atECM2-1b*	At2g29580	2	10		>1-2a	RRM, 1;CCCH, 1;	
		*atECM2-2*	At5g07060	5				CCCH, 1;	
		
KIAA0560		*atAquarius*	At2g38770	2	11				
		
MGC23918		*atMGC23918*	At3g05070	3	7				
		
G10	Cwc14p	*atG10*	At4g21110	4	12			G10, 1;	
		
Cyp E		*atCypE1a/CYP2*	At2g21130	2	4		>2-4c	Pro_isomerase, 1	
		*atCypE1b*	At4g38740	4	59		>2-4c	Pro_isomerase, 1;	
		*atCypE2a/ROC3*	At2g16600	2	39		>2-4a	Pro_isomerase, 1	
		*atCypE2b*	At4g34870	4	80		>2-4a	Pro_isomerase, 1;	
		
PPIase-like 1		*atPPIase-like1*	At2g36130	2	10			Pro_isomerase, 1;	

									
**2.5 Proteins specific for BΔU1**									
NPW38		*atNPW38*	At2g41020	2	16	AltD (1); IntronR (1);		WW, 2;	
		
N-CoR1		*atN-CoR1*	At3g52250	3	3			SANT, 2;Homeodomain_like, 2;	
		
hPrp4 kinase		*atPRP4K-1*	At3g25840	3	13	ExonS (1);		Pkinase, 1;TyrKc, 1;S_Tkc, 1;, 1;Kinase_like, 1;	
		*atPRP4K-2*	At1g13350	1	5	IntronR (1);		Pkinase, 1;TyrKc, 1;S_Tkc, 1;, 1;Kinase_like, 1;	
		*atPRP4K-3*	At3g53640	3				Pkinase, 1;TyrKc, 1;S_Tkc, 1;, 1;Kinase_like, 1;	
		
FBP-21		*atFBP21*	At1g49590	1	12	ExonS (1); IntronR (3);	C2H2, 1;		
		
TBL1-rp 1		*atTBL1-rp1*	At5g67320	5	14			WD-40, 5;Peptidase_S9A_N, 1;LisH, 1;WD40like, 1;	
		
Smc-1		*atSmc1*	At3g54670	3	12			ATP_GTP_A_BS, 1;SMC_N, 1;SMC_C, 1;ABC_transporter, 1;SMC_hinge, 1;	

									
**2.6 Exon junction complex (EJC) proteins**									
ALY	Yra1p	*atALY-1a*	At5g02530	5	19	IntronR (1);		RRM, 1;	
		*atALY-1b*	At5g59950	5	16	IntronR (1);		RRM, 1;	
		*atALY-2a*	At5g37720	5	17		>1-5b	RRM, 1;	
		*atALY-2b*	At1g66260	1	38	ExonS (1);	>1-5b	RRM, 1;	
		
Y14		*atY14*	At1g51510	1	10	IntronR (1);		RRM, 1;RBM8, 4;	
		
Srm160-like		*atSRM102*	At2g29210	2	18	AltA (1);		PWI, 1	
		
Magoh		*atMagoh*	At1g02140	1	19			Mago_nashi, 1;	
		
Nuk-34/eIF4A3/DDX48		*atDDX48/eIF4A3-1*	At3g19760	3	50		>1-3a	DEAD, 1;Helicase_C, 1;	
		*atDDX48/eIF4A3-2*	At1g51380	1	5		>1-3a	DEAD, 1;Helicase_C, 1;	
		
RNPS1		*atSR45/atRNPS1*	At1g16610	1	27	AltA (1);		RRM, 1	[63]
		
UAP56		*atUAP56a*	At5g11200	5	21	AltA (1);		DEAD, 1; Helicase_C, 1	
		*atUAP56b*	At5g11170	5	25			DEAD, 1; Helicase_C, 1	
		
pinin		*atPinin*	At1g15200	1	9	AltA (1);		Pinin/SDK/memA, 1;	

									
**2.7 Second step splicing factors**									
Prp22	Prp22	*atPrp22-1*	At3g26560	3	11			DEAD, 1; Helicase_C, 1; S1, 1; HA2, 1;	
		*atPrp22-2*	At1g26370	1	5			DEAD, 1; Helicase_C, 1; HA2, 1	
		*atPrp22-3*	At1g27900	1	15			DEAD, 1; Helicase_C, 1; HA2, 1	
		
Prp17	Prp17p	*atPrp17-1*	At1g10580	1	10			WD-40, 7;	
		*atPrp17-2*	At5g54520	5	5	AltA (1);		WD-40, 6;	
		
Prp18	Prp18	*atPrp18-1*	At1g03140	1	16			Prp18, 1; SFM 1;	
		*atPrp18-2*	At1g54590	1				Prp18, 1	
		
Slu7	Slu7p	*atSLU7-1a*	At1g65660	1	6				
		*atSLU7-1b*	At4g37120	4	11				
		*atSLU7-2*	At3g45950	3					
		
Prp16	Prp16p	*atPrp16*	At5g13010	5	22			DEAD, 1; Helicase_C, 1; HA2, 1	

									
**2.8 Other known splicing factors**									
SRm300		*atSRM300like*	At3g23900	3	5	AltD (1);		RRM, 1; Filamin/ABP280 repeat, 1	
		
hTra-2/SFRS10		*atTra/SFRS1*	At1g07350	1	25	ExonS (1); IntronR (3);		RRM, 1	
		
Prp2		*atPrp2-1a*	At1g32490	1	9		>1-2c	DEAD, 1; Helicase_C, 1; HA2, 1	
		*atPrp2-1b*	At2g35340	2			>1-2c	DEAD, 1; Helicase_C, 1; HA2, 1	
		*atPrp2-2*	At4g16680	4				DEAD, 1; Helicase_C, 1; HA2, 1	
		
Prp5		*atPrp5-1a*	At3g09620	3				DEAD, 1; Helicase_C, 1	
		*atPrp5-1b*	At1g20920	1	11			DEAD, 1; Helicase_C, 1	
		*atPrp5-2*	At2g47330	2	9			DEAD, 1; Helicase_C, 1	
		
hDbr1	dbr1	*atDbr1*	At4g31770	4	12			Metallophos, 1; DBR1, 1	

									
**3.1 SR protein kinase**									
Lammer/CLK kinase		*AFC1*	At3g53570	3	11	AltA (1); IntronR (3);		PKinase, 1; TyrKc, 1; S_Tkc, 1; PKinase-like, 1	[74]
		*AFC2*	At4g24740	4	9	ExonS (1);		PKinase, 1; TyrKc, 1; S_Tkc, 1; PKinase-like, 1	[74]
		*AFC3*	At4g32660	4	9	AltD (1); IntronR (1);		PKinase, 1; TyrKc, 1; S_Tkc, 1; PKinase-like, 1	[74]
		
SRPK1		*atSRPK1a*	At2g17530	2	7		>2-4a	PKinase, 1; TyrKc, 1; S_Tkc, 1; PKinase-like, 1	
		*atSRPK1b*	At4g35500	4	10		>2-4a	PKinase, 1; TyrKc, 1; S_Tkc, 1; PKinase-like, 1	
		
SRPK2		*atSRPK2a*	At5g22840	5	2			PKinase, 1; TyrKc, 1; S_Tkc, 1; PKinase-like, 1	
		*atSRPK2b*	At3g53030	3	7			PKinase, 1; TyrKc, 1; S_Tkc, 1; PKinase-like, 1	
		*atSRPK2c*	At3g44850	3	1			PKinase, 1; TyrKc, 1; PKinase-like, 1	

									
**3.2 Glycine-rich RNA binding protein**									
HnRNP A/B		*atGRBP1a*	At1g18630	1	5		>1-1c	RRM, 1	
		*atGRBP1b*	At1g74230	1	14		>1-1c	RRM, 1; Eggshell, 4	
		*atGRBP1c*	At4g13850	4	17		>3-4	RRM, 1	
		*atGRBP1d*	At3g23830	3	12	AltA (1);	>3-4	RRM, 1	
		*atGRBP1e*	At5g61030	5	8			RRM, 1; PfkB_Kinase, 1	
		*atGRBP2*	At2g16260	2				RRM, 1	
		*AtGRP7/atGRBP3a*	At2g21660	2	182	AltD (1); IntronR (3);	>2-4c	RRM, 1	[77]
		*AtGRP8/atGRBP3b*	At4g39260	4	67	AltB (1); AltD (1); IntronR (5);	>2-4c	RRM, 1	[77]

									
**3.3 hnRNP A/B family**									
hnRNP A/B		*AtRNPA/B_1*	At4g14300	4	4			RRM, 2	[11]
		*AtRNPA/B_2*	At2g33410	2	13			RRM, 2	[11]
		*AtRNPA/B_3*	At5g55550	5	13	IntronR (3);	>4-5c	RRM, 2	[11]
		*AtRNPA/B_4*	At4g26650	4	21		>4-5c	RRM, 2	[11]
		*AtRNPA/B_5*	At5g47620	5	12	AltD (2);		RRM, 2	[11]
		*AtRNPA/B_6*	At3g07810	3	18	AltA (1);		RRM, 2; FKBP_PPIASE_2, 2	[11]
		*AtRNPA/B_7*	At1g58470	1	6			RRM, 2	
		*AtRNPA/B_8a*	At5g40490	5	3			RRM, 2; Eggshell, 4	
		*AtRNPA/B_8b*	At1g17640	1				RRM, 2	
		*AtRNP_N1*	At3g13224	3	16	IntronR (1);		RRM, 2;HUDSXLRNA, 2;	
		*UBA2a*	At3g56860	3	23	IntronR (1);	>2-3	RRM, 2	[78]
		*UBA2b*	At2g41060	2	9		>2-3	RRM, 2	[78]
		*UBA2c*	At3g15010	3	10	IntronR (1);		RRM, 2	[78]

									
**3.4 Other hnRNPs (with animal homologs)**									
hnRNP E1/E2		*at-hnRNP-E*	At3g04610	3	10			KH, 3;	
		
hnRNP F/ hnRNP H		*at-hnRNP-F/ AtRNPH/F_1*	At5g66010	5	9	AltA (1);		RRM, 2	[11]
		*at-hnRNP-H/ AtRNPH/F_2*	At3g20890	3				RRM, 2	[11]
		
hnRNP G		*at-hnRNP-G1*	At5g04280	5	6			RRM, 1; CCHC, 1	
		*at-hnRNP-G2*	At3g26420	3	35	AltA (1);		RRM, 1; CCHC, 1	
		*at-hnRNP-G3*	At1g60650	1	7			RRM, 1; CCHC, 1	
		
hnRNP P2		*at-hnRNP-P*	At1g50300	1	9			RRM, 1; ZnF_RBZ, 2	
		
hnRNP R/Q		*hnRNP-R1*	At4g00830	4	19			RRM, 3;	
		*hnRNP-R2*	At3g52660	3	1		>2-3	RRM, 3;	
		*hnRNP-R3 / AtRNPA/B_9*	At2g44710	2	13		>2-3	RRM, 3	
		
CUG-BP		*AtCUG-BP1*	At4g03110	4	4	AltA (1); IntronR (1);		RRM, 3; HUDSXLRNA, 4	[11]
		*AtCUG-BP2*	At1g03457	1	9	AltA (1);		RRM, 3; HUDSXLRNA, 4	[11]
		
(CUG-BP)		*atFCA1*	At4g16280	4	13	AltB (1); IntronR (1);		RRM, 2; WW, 1	[81]
		*atFCA2*	At2g47310	2	6			RRM, 2; WW, 1	

									
**3.5 Other plant hnRNPs**									
		*AtUBP1a*	At1g54080	1	48	AltA (1);	>1-3b	RRM, 3	[84]
		*AtUBP1c*	At3g14100	3	13		>1-3b	RRM, 3	[84]
		*AtUBP1b*	At1g17370	1	17			RRM, 3	[84]
		
		*UBA1a*	At2g22090	2	15		>2-4c	RRM, 1	[78]
		*UBA1b*	At2g22100	2	2		>2-4c	RRM, 1	[78]
		*UBA1c*	At2g19380	2	1			RRM, 1; C2H2, 3	[78]
		
		*atRBP45a*	At5g54900	5	42		>4-5c	RRM, 3	[85]
		*atRBP45c*	At4g27000	4	52		>4-5c	RRM, 3	[85]
		*AtRBP45b*	At1g11650	1	53			RRM, 3	[85]
		*atRBP45d*	At5g19350	5	10			RRM, 3	
		
		*AtRBP47a*	At1g49600	1	10		>1-3a	RRM, 3	[85]
		*AtRBP47b*	At3g19130	3	21		>1-3a	RRM, 3	[85]
		*AtRBP47c*	At1g47490	1	23	IntronR (1);		RRM, 3	[85]
		*AtRBP47c'*	At1g47500	1	12			RRM, 3	[85]
		
		*Ath1*	At4g16830	4	34			HANP4_PAI-RBP1, 1	[105]
		*Ath2*	At4g17520	4	29		>4-5a	HANP4_PAI-RBP1, 1	[105]
		*Ath3*	At5g47210	5	67	IntronR (1);	>4-5a	HANP4_PAI-RBP1, 1	

Gene names were kept consistent with names used in previous publications or derived from the names of the respective homologs (yeast names are given in the S.c. column, where available). The Tnb column gives the numbers of cognate cDNAs and ESTs supporting the gene structure. The AltS column indicates evidence for alternative splicing, including alternative donor site (AltD), alternative acceptor site (AltA), alternative position (AltP, both acceptor and donor sites are different), exon skipping (ExonS), and intron retention (IntronR). Chromosomal duplication indicates a known chromosome duplication region. Functional groups of proteins are separated by long lines spanning all columns. Different members in the group are separated by short lines starting at the *Arabidopsis *gene name. Genes duplicated in *Arabidopsis *are clustered together with no line between them. Dash line separate the Prp19 complex from other 35S U5 associated proteins and * indicates proteins in that complex. Abbreviations for domains are as follows: ABC_transporter: ABC transporter; Armadillo: Armadillo; ARM: ARM repeat fold; ATP_GTP_A_BS: ATP/GTP-binding site motif A (P-loop); BCAS2: Breast carcinoma amplified sequence 2; C2H2: Zn-finger, C2H2 matrin type; C2H2: Zn-finger, C2H2 type; CCCH: Zn-finger, C-x8-C-x5-C-x3-H type; CCHC: Zn-finger, CCHC type; CPSF_A: CPSF A subunit, C-terminal; Cwf_Cwc_15: Cwf15/Cwc15 cell cycle control protein; DBR1: Lariat debranching enzyme, C-terminal; DEAD: ATP-dependent helicase, DEAD-box; DEAD: DEAD/DEAH box helicase; DIM1: Pre-mRNA splicing protein; DUF259: Protein of unknown function DUF259; DUF382: Protein of unknown function DUF382; EFG_C: Elongation factor G, C-terminal; EFG_IV: Elongation factor G, domain IV; Eggshell: Eggshell protein; FF: FF domain; FKBP_PPIASE_2: Peptidylprolyl isomerase, FKBP-type; G10: G10 protein; GTP_EFTU_D2: Elongation factor Tu, domain 2; GTP_EFTU: Protein synthesis factor, GTP-binding; HA2: Helicase-associated region; HANP4_PAI-RBP1, 1: Hyaluronan/mRNA binding protein; HAT: RNA-processing protein, HAT helix; Helicase_C: Helicase, C-terminal; Homeodomain_like: Homeodomain-like; Hsp70: Heat shock protein Hsp70; HUDSXLRNA: Paraneoplastic encephalomyelitis antigen; Isy1: Isy1-like splicing; Kinase_like: Protein kinase-like; LisH: Lissencephaly type-1-like homology motif; LRR: Leucine-rich repeat,; Mago_nashi: Mago nashi protein; Metalloph: Metallophosphoesterase; MIF4G: Initiation factor eIF-4 gamma, middle; Mov34: Mov34/MPN/PAD-1; mrCtermi: Molluscan rhodopsin C-terminal tail; Nop: Pre-mRNA processing ribonucleoprotein, binding region; Peptidase_S9A_N: Peptidase S9A, prolyl oligopeptidase, N-terminal beta-propeller domain; PfkB_Kinase: Carbohydrate kinase, PfkB; PHOSPHOPANTETHEINE: Phosphopantetheine attachment site; Pinin/SDK/memA: Pinin/SDK/memA protein; Pkinase: Protein kinase; Pro_isomerase: Peptidyl-prolyl cis-trans isomerase, cyclophilin type; Prp18: Prp18 domain; Prp1_N: PRP1 splicing factor, N-terminal; PSP: PSP, proline-rich; PWI: Splicing factor PWI; RBM8: RNA binding motif protein 8; Ribosomal_L7Ae: Ribosomal protein L7Ae/L30e/S12e/Gadd45; RPR: Regulation of nuclear pre-mRNA protein; RRM: RNA-binding region RNP-1 (RNA recognition motif); S1: RNA binding S1; SANT: Myb DNA-binding domain; SAP_155: Splicing factor 3B subunit_1; SART-1: SART-1 protein; Sec63: Sec63 domain; SF3b10: Splicing factor 3B subunit 10; SFM: Splicing factor motif; SKIP/SNW: SKIP/SNW domain; Small_GTP: Small GTP-binding protein domain; SMC_C: Structural maintenance of chromosome protein SMC, C-terminal; SMC_hinge: SMCs flexible hinge; SMC_N: SMC protein, N-terminal; Sm_like_riboprot: Small nuclear-like ribonucleoprotein; Sm: Small nuclear ribonucleoprotein (Sm protein); S_Tkc: Serine/threonine protein kinase; Surp: SWAP/Surp; Thioredoxin_2: Thioredoxin domain 2; TPRlike: TPR-like; TPR: TPR repeat; Tudor: Tudor domain; TyrKc: Tyrosine protein kinase; Ubox: Zn-finger, modified RING; UCH: Peptidase C19, ubiquitin carboxyl-terminal hydrolase family 2; UPF0123: Protein of unknown function UPF0123; UPF0123: Protein of unknown function UPF0123; WD-40: G-protein beta WD-40 repeat; WD40like: WD40-like; WW: WW/Rsp5/WWP domain; ZnF_RBZ: Zn-finger, Ran-binding; ZnF_U1: Zn-finger, U1-like; ZnF_UBP: Zn-finger in ubiquitin thiolesterase.

**Table 3:** Duplication source involving *Arabidopsis *splicing-related proteins

	Genes	Family*	Single/multiple	Duplication ratio	Duplication events	Chromosomal duplications	Chromosomal duplication ratio
snRNP proteins	91	54	27/27	50.0%	37	7	18.9%
Splicing factors	109	58	33/25	43.1%	51	14	27.5%
Splicing regulator	60	18	4/14	77.8%	42	11	26.2%
Total	260	130	64/66	50.8%	130	32	24.6%

*Family indicates both single copy gene and multiple-copy gene families. The Chromosomal duplication ratio column gives the fraction of all duplication events caused by chromosomal duplications.

**Table 4:** Alternative splicing in splicing-related genes

	Genes	AltA	AtlD	AltP	ExonS	IntronR	Overall	Ratio
snRNP proteins	91	6	3	1	3	11	22	23.2%
Splicing factors	109	14	5	0	11	21	38	34.9%
Splicing regulator	60	8	4	2	1	12	20	33.3%
Total	260	28	12	3	15	44	80	30.8%

The column entries are the numbers of genes in which the respective alternative splicing events can occur. AltA, alternative acceptor site; AltD, alternative donor site; AltP, alternative intron position (both acceptor and donor sites are different); ExonS, exon skipping; IntronR, intron retention. The Overall and Ratio columns give the number and fraction of genes with any type of alternative splicing, respectively.
